# Laboratory protocol is important to improve the correlation between target copies and metabarcoding read numbers of seed DNA in ground beetle regurgitates

**DOI:** 10.1038/s41598-023-29019-8

**Published:** 2023-02-03

**Authors:** Veronika Neidel, Michael Traugott

**Affiliations:** Applied Animal Ecology Research Unit, Department of Zoology, University of Innsbruck, Technikerstraße 25, 6020 Innsbruck, Austria

**Keywords:** Agroecology, Ecological networks, Ecosystem ecology, Ecosystem services

## Abstract

DNA metabarcoding is increasingly important for studying feeding interactions, yet it remains unresolved whether reporting read counts or occurrences is to be preferred. To address this issue for gut content samples, basic experimental data on the relationship between read numbers and initial prey DNA amounts and how both change over digestion time is needed. Using regurgitates of the carabid *Pseudoophonus rufipes* the digestion of *Taraxacum officinale* seeds was documented for 128 h post-feeding to determine how the number of (1) seed DNA copies and (2) metabarcoding reads change over digestion time, and (3) how they correlate to each other. Additionally, we tested (4) whether PCR cycle-numbers during library preparation affect this correlation. The number of copies and reads both decreased with digestion time, but variation between samples was high. Read and copy numbers correlated when using a library preparation protocol with 35 cycles (R^2^ = 42.0%), yet a reduction to 30 cycles might significantly improve this correlation, as indicated by additional PCR testing. Our findings show that protocol optimization is important to reduce technical distortions of read numbers in metabarcoding analysis. However, high inter-sample variation, likely due to variable digestive efficiency of individual consumers, can blur the relationship between the amount of food consumed and metabarcoding read numbers.

## Introduction

Over the past decade, the use of DNA metabarcoding for the study of feeding interactions has increased considerably^1^. DNA metabarcoding (hereafter simply, ‘metabarcoding’) is a method that uses DNA barcodes for the simultaneous identification of multiple species contained in mixed samples, typically via high-throughput sequencing (HTS) techniques^2–4^. For each taxon detected within a dietary sample, a specific number of sequence reads is generated during HTS, which has prompted the idea that the number of reads and the proportion of reads between taxa could potentially hold information on the biomass or number of prey items as well as the relative prey proportions ingested by a consumer^5^. Currently, there is an ongoing discussion whether metrics based on read numbers are indeed superior to mere occurrences (presence/absence data) when reporting the results of metabarcoding studies of trophic interactions^6^. For faecal samples specifically the aspect of quantification has been addressed with ambiguous results^7^, however, there is a striking lack of information regarding the significance of read numbers for samples based on gut content.

Yet, gut content, including dissected guts, whole-body extracts (homogenized individuals) and regurgitates, plays an important role for molecular diet analysis, especially when it comes to the study of arthropods’ feeding interactions^8–10^. Metabarcoding analysis of gut content samples is considered more challenging than of faecal samples, due to the effect of unknown digestion times on recoverable prey DNA.

In studies using diagnostic PCR assays, the effect of digestion time on prey DNA is taken into account by reporting the presence/absence data translated to detectability at specific times after feeding^11–13^ or through detectability half-lives^14,15^. Generally, the detectability of prey DNA in the gut content of a consumer decreases over digestion time, suggesting a continuous breakdown of DNA. To study how the copy numbers in predators’ guts are affected by prey numbers and digestion time, diagnostic gut content analysis has been done with quantitative or real-time PCRs (qPCR). For example, prey-specific DNA target molecules of the silverleaf whitefly *Bemisia tabaci* were shown to increase linearly with the number of consumed individuals and to decrease over time of digestion in the guts of the ladybird beetle *Propylea japonica*^16^. Prey DNA in the guts of the ladybird beetle *Coleomegilla maculata* was found to decrease exponentially, but with different decay rates for different prey items^17^. An exponential decay over digestion time was also reported for the number of reads in a shotgun sequencing study using dissected guts of ladybird beetles fed with aphids^18^. For metabarcoding assays, however, no studies are available that report on the amount of prey DNA in gut content samples, its change over time of digestion and how these values are related to read numbers of metabarcoding analysis.

Here, we use a set of regurgitate samples of the omnivorous ground beetle *Pseudoophonus rufipes* De Greer, 1774 (Coleoptera: Carabidae), that map the first 128 h of digesting seeds of *Taraxacum officinale* (Asteraceae), across 11 points in time after feeding. For this sample set, we determine how (1) the number of DNA target copies contained in DNA extracts, and (2) the number of reads, produced by a standard metabarcoding protocol, change over digestion time, and (3) how they correlate to each other. Initial copy numbers were measured with droplet digital PCR (ddPCR), which provides a direct measure of copy numbers for each sample^19^. In environmental monitoring surveys, ddPCR was found to be more sensitive^20^, and cheaper than qPCR^21^, and at the same time, equally accurate^21,22^.

Further, we (4) compare the copy- and read-numbers to the relative target-fragment amount in the library-PCR products, which can be viewed as a check-point between the DNA extracts and the HTS-output. Therefore, we combined end-point PCRs with capillary electrophoresis (celPCRs). During capillary electrophoresis, the signal strength of each PCR product is estimated in relative fluorescent units (RFU), a measure that strongly correlates with the initial copy numbers in DNA extracts^23^.

Based on the observed distortions in copy numbers, we conducted additional PCR assays to study, if (5) increasing or lowering the PCR cycle number can mitigate amplification biases during library preparation and provide a means for getting better estimations of initial target DNA copy numbers.

## Results

### Prey DNA copy numbers vary strongly between samples, means decrease over time of digestion

**Figure 1:**
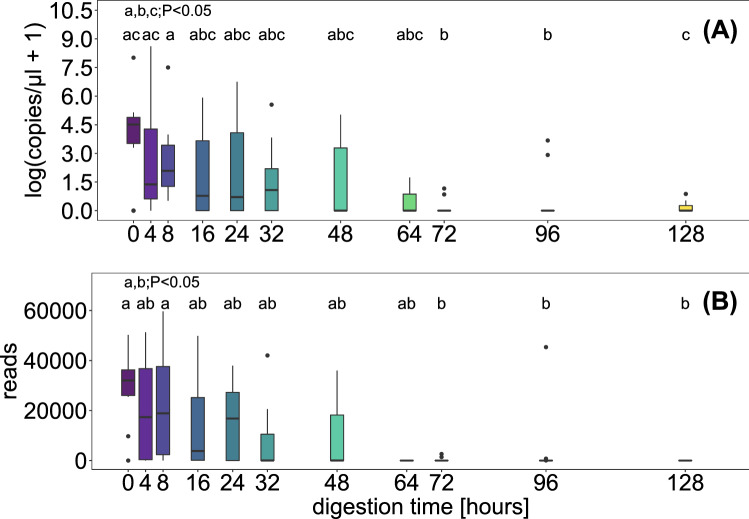
Target copies/µl DNA extract measured with droplet digital PCR (**A**, n = 111) and read numbers obtained in HTS (**B**, n = 102) of the plant-specific *trn*L c-h target fragment in regurgitates of *Pseudoophonus rufipes*, collected at 0, 4, 8, 16, 24, 32, 48, 64, 72, 96 and 128 h after feeding on three seeds of *Taraxacum officinale.* Boxplots of measured values, letters indicate time groups that were significantly different in pairwise Wilcoxon tests with BH correction for multiple comparisons.

Absolute copy concentrations of the *trn*L c-h target fragment in DNA extracts of regurgitates from *P. rufipes* after the consumption of three *T. officinale* seeds ranged from 0.65 to 5490 copies/µl extract. The mean copy numbers per time point were found to decrease with digestion time (n = 111: Pearson r = − 0.437, 95% CI [− 0.577, − 0.273], p < 0.001, Fig. 1A). The standard deviation, however, was quite high and took values that were 1.4–2.9 times the mean value (Supplementary Table S3). Overall, there was a significant difference in copies measured at the different times after feeding (Kruskal–Wallis chi-squared = 31.187, df = 10, p < 0.001). In pairwise comparisons of the individual time groups, no significant difference between the average copy numbers measured between 0 and 64 h after feeding was found. Average copy numbers at 72 and 96 were significantly lower than those measured at 0, 4 and 8 h after feeding (Fig. 1 and Supplementary Table S5a).

### Decrease of prey DNA reads over time of digestion

We recovered between 7 and 59,968 sequence reads per sample that matched a reference database entry at a level of 98% and above (mean ± sd = 26,256.5 ± 15,964.7 reads). The resulting species identities indicated that the targeted fragment of the *trn*L *c-h* marker region did not allow for species-level determination: only a fraction of the recovered sequences was identified as *T. officinale,* the seed provided to the beetles as an experimental diet (mean 0.03% of sequences per sample with match ≥ 98% for *T. officinale*). Of the total reads assigned to the target fragment length of 135–160 bp, 23 plant-families were detected, of which 87.6% were assigned to be Asteraceae, all other families made up 3.6% or less of the total read count over all samples (Table S6, Figure S1). Per sample, a mean of 78.6 ± 37.3% of the sequences were assigned to the family Asteraceae, including the afore mentioned ones assigned to *T. officinale*. Thus, we assumed these reads to be the result of the consumed seeds and used the respective read counts as a reference value for analysis. The Asteraceae reads were negatively correlated with the digestion time (Pearson r = − 0.465, 95% CI [− 0.605; 0.298], p < 0.001). As with copy numbers, we observed high variability in measured reads between samples at the same time after feeding on three seeds of *T. officinale* (Supplementary Table S4). There was a significant difference in Asteraceae reads measured at different times after feeding (Kruskal–Wallis chi-squared = 34.689, df = 10, p < 0.001), and pairwise comparison revealed a significant difference between read numbers at 0 and 12 h compared to 72 h and longer (Fig. 1B, Supplementary Table S5b).

### Initial target copy numbers were correlated with HTS-read count

**Figure 2:**
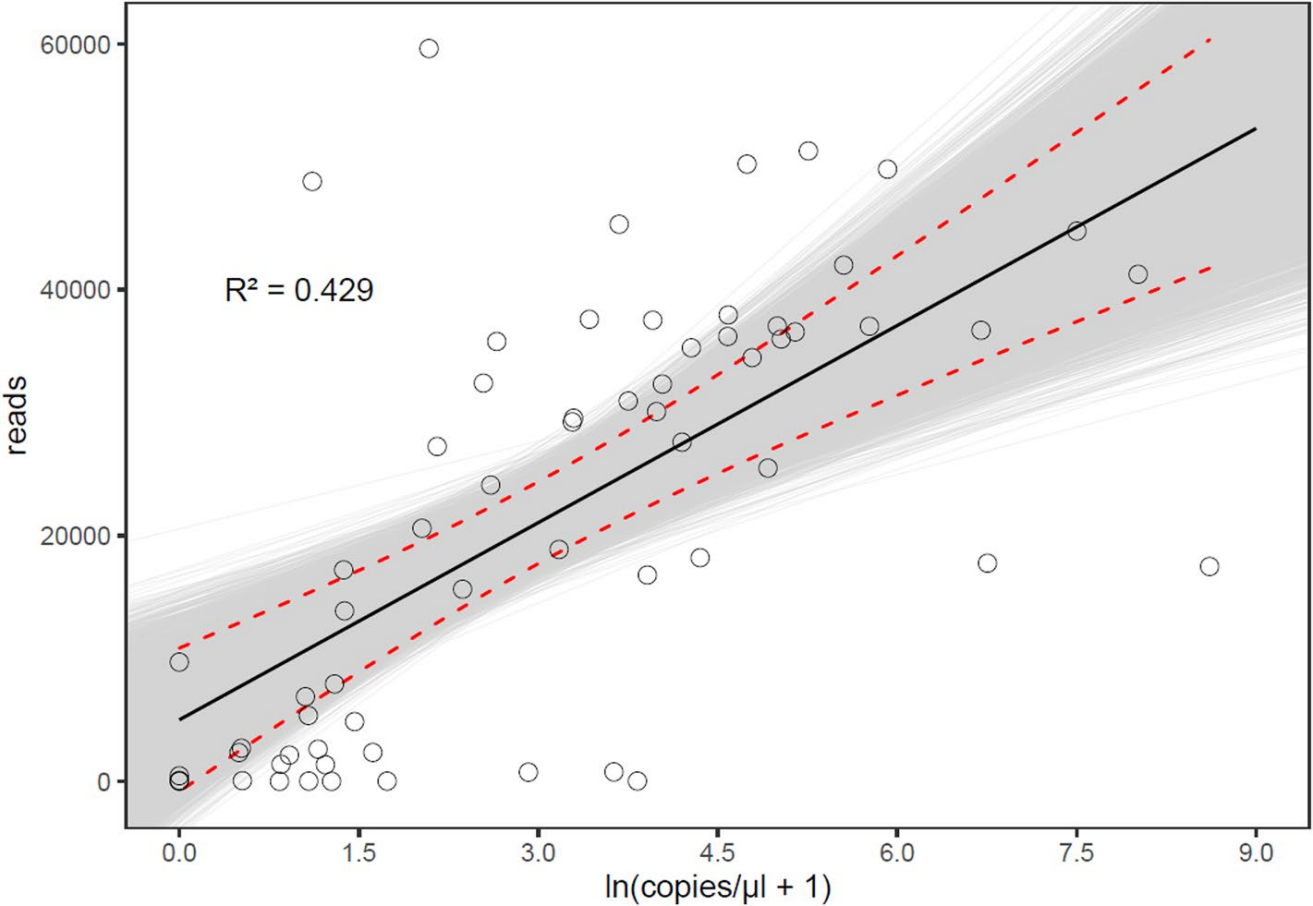
Correlation of copies/µl and read numbers of the plant-specific *trn*L c-h target fragment in regurgitates of *Pseudoophonus rufipes* collected at 0, 4, 8, 16, 24, 32, 48, 64, 72 and 96 h after feeding on 1–3 seeds of *T. officinale.* Dots in the scatterplot (○) are measured values, black bold lines are predicted values of a linear regression model of copies and reads, dashed lines are 95% confidence intervals, shaded area marks boot-strapped 95% confidence interval, and R^2^ indicates goodness of model-fit.

The linear model including log(copies/µl + 1) as a predictor of the recovered read numbers could explain 42.0% of the observed variation in the sequence reads assigned to the family Asteraceae in our HTS analysis (R^2^ = 0.43, F (1, 62) = 46.6, p < 0.001; Fig. 2). A 1% increase in copy numbers was predicted to lead to an increase of 53.5 reads (95% CI [33.1, 68.2], p < 0.001, Supplementary Table S1A).

### Signal strengths of library PCR correlated to read counts and initial target copy number

**Figure 3:**
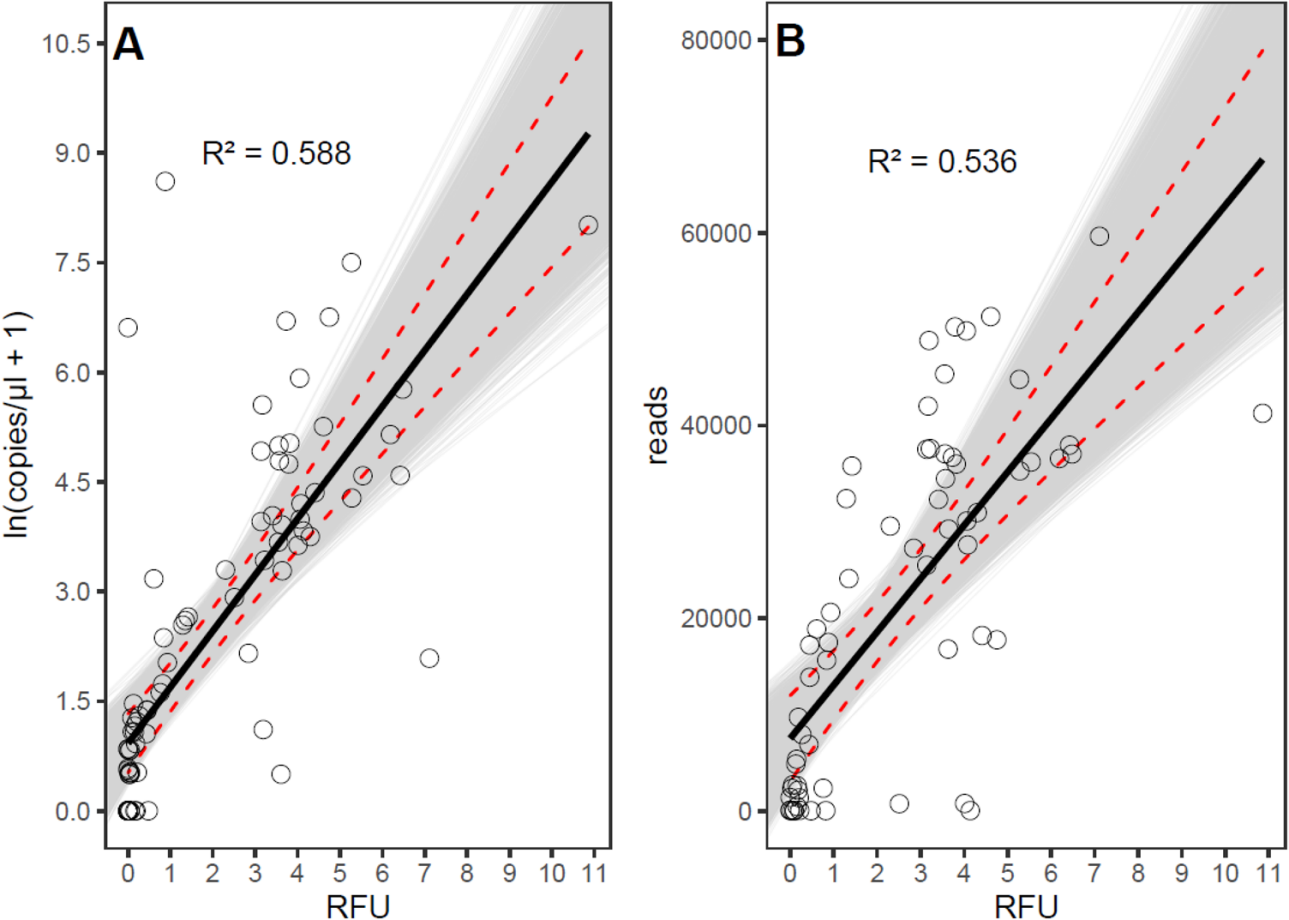
Signal strengths [RFU] of celPCR products of the library preparation PCR for high-throughput sequencing, plotted against (**A**) copies/µl and (**B**) *Asteraceae* read numbers of the plant-specific *trn*L *c-*h target fragment, measured in regurgitates of *Pseudoophonus rufipes*, collected at 0, 4, 8, 16, 24, 32, 48, 64, 72, and 96 h after feeding on 1–3 seeds of *Taraxacum officinale.* In the scatterplots, dots (○) represent measured values, black bold lines are predicted values of linear regression models, dashed lines are 95% confidence intervals, shaded area marks boot-strapped 95% confidence interval, and R^2^ indicates goodness of model-fit.

When modelling the copy numbers from the library preparation PCR product signal strengths, the linear regression explained 58.8% of the observed variation (R^2^ = 0.588, F (1, 86) = 122.9, p < 0.001, Supplementary Table S2). Copy numbers increase by 11.5% with every 0.1 increase in RFU-values (95%CI [8.3, 15.0], p < 0.001, Fig. 3A). The model prediction of read numbers from the signal strengths of the PCR product [RFU] was also statistically significant (R^2^ = 0.536, F (1,62) = 71.61, p < 0.001). The read numbers increase by 553.4 reads with every 0.1 increase in RFU-values (95% CI [395.9, 697.7], p < 0.001, Fig. 3B, Supplementary Table S1B).

### Reduced reaction volume in PCR yields a better estimation of initial target copy numbers

**Figure 4:**
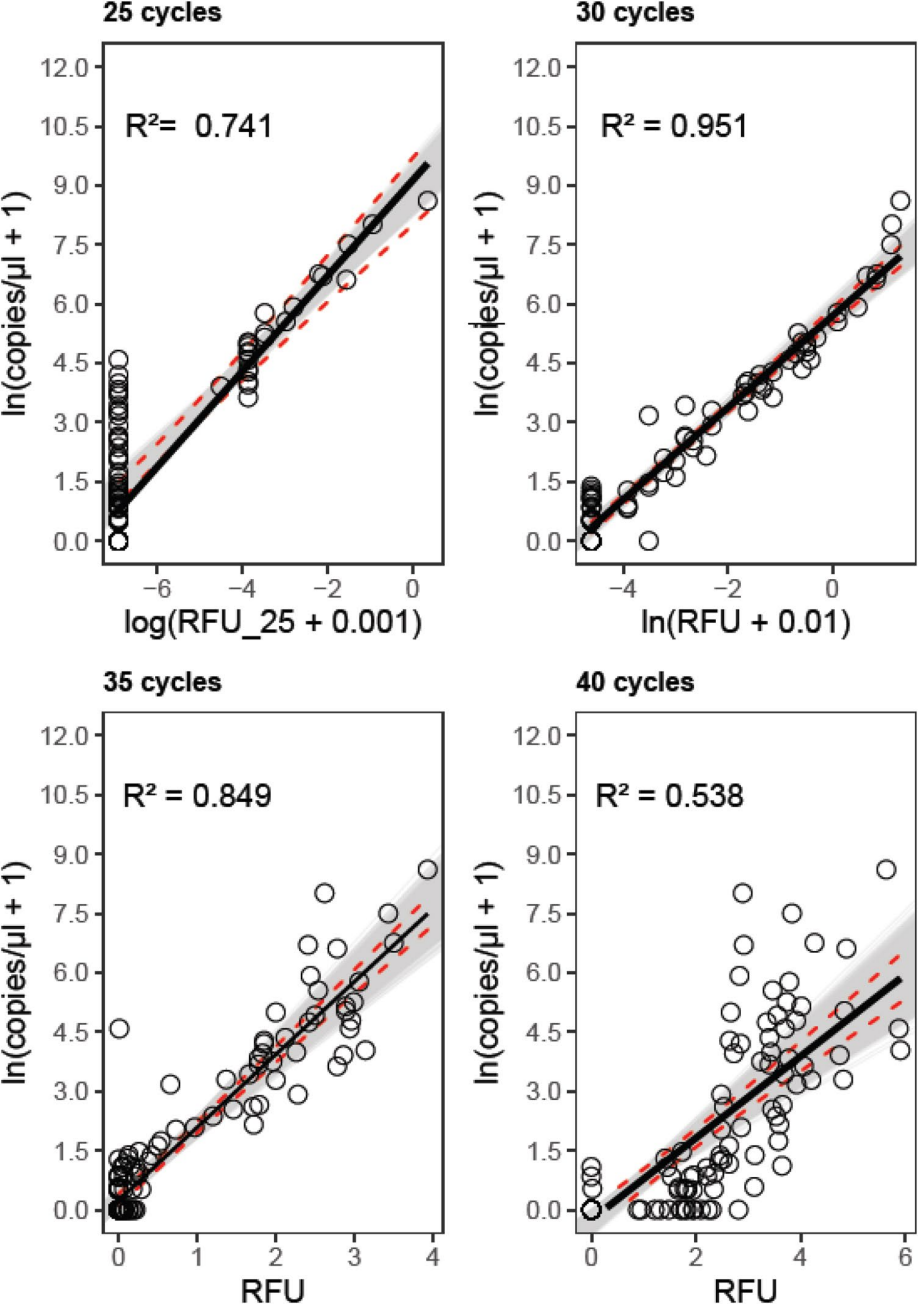
Correlations of plant-specific *trn*L-target copies/µl DNA extract and signal strength (RFU) of celPCRs products after 25, 30, 35 or 40 cycles with a reaction volume of 10 µl. Dots (○) are measured values, black bold lines are predicted values of linear regression models, dashed lines are 95% confidence intervals, shaded area are boot-strapped 95% confidence intervals and R^2^ indicates goodness of model-fit.

RFU-values of singleplex PCRs with a volume of 10 µl and 35 cycles had a greater explanatory power for the initial copy numbers (Fig. 4, Supplementary Table S2D), than the library preparation PCR with a reaction volume of 15 µl and 35 cycles (Fig. 3B, Supplementary Table S2A). Variation in copy numbers was explained to 84.9% by RFU-values as predictor (R^2^ = 0.849, F (1, 90) = 504.1, p < 0.001). Copy numbers increased by 48.45% with every 0.1 RFU increase (95% CI [38.6, 59.7], p < 0.001).

### PCR cycle number strongly affects the correlation between RFUs and initial copy numbers

The increase and decrease of cycles also changed the relationship between the copy numbers and the PCR product signal strengths (RFU). While all tested PCR cycle variants reflected the initial copies well, there was a varying degree of explanatory power (Fig. 4). The model with RFUs derived from celPCRs with 30 cycles showed the strongest correlation between RFU-values and copy numbers (R^2^ = 0.95, F (1, 90) = 1760, p < 0.001). RFU-values obtained from celPCRs with 25 cycles explained the variation to 73.9% (F(1, 90) = 258, p < 0.001). The model for RFUs after 40 cycles could account for only 53.3%, (F(1, 90) = 104.9, p < 0.001) and had, thus, the lowest explanatory power of the assessed thermocycling protocols.

## Discussion

We measured the absolute target copy numbers and the HTS read numbers of a plant-specific DNA marker in regurgitate samples of the carabid *P. rufipes* at different times after feeding on dandelion seeds to study its change during digestion. The intuitive assumption, that target DNA copy numbers would be high early, and low late during the digestive progress, was only partly confirmed by our results. The mean copy numbers in samples collected at the same time after feeding indeed decreased with increasing digestion time due to a shrinking number of samples with positive detections. Moreover, we found that read numbers from metabarcoding were only moderately correlated to the target copy numbers contained in the original DNA extracts. Thus, on the level of the individual sample, neither copy numbers nor HTS read numbers of gut content samples held reliable information on whether a feeding event had taken place very recently before sampling. This, once more, stresses the need to maximise biological replication in molecular diet studies^24^. While some samples failed to amplify DNA even soon after feeding, a high number of target copies was more frequently associated with a shorter digestion time. Thus, if high read counts occur in a major part of the tested individuals, a prey item is likely to be consumed frequently and thus be of importance to the consumer species. Consequently, we suggest, that when working with gut content samples of arthropods for the study of feeding interactions with plant seeds, sufficient samples numbers need to be collected to capture differences between field sites or consumers.

While we did not compare different meal sizes directly, our results indicate that meals of the same size ingested by different consumer individuals of the same taxon can yield a wide range of different target copy numbers, and further read numbers in HTS. For whole-body samples of ladybird beetles, Weber and Lundgren^17^ found that prey DNA means estimated for prey items increased with an increasing number of items consumed. Yet, the data had wide deviation within the meal size variants, leading to a strong overlap among them, which was also reflected in the prey DNA copy numbers over time of digestion. Zhang et al.^16^ reported a linear relationship between estimated copy numbers and ingested number of larvae, however, SDs were not reported. Both studies used animals as experimental prey, while we fed consumers with plant seeds. For gut content samples of carabids, the food DNA detection probability curves over time of digestion follow a constant decrease for animal prey but less so for plant seeds^25^, which might also be the case regarding copy numbers.

Several technical, methodological and biological factors can cause variation between samples, for example extraction efficiency^5,26^ or PCR inhibition^27,28^. For the present study, DNA was extracted from regurgitate samples using an automated extraction platform with a magnetic-bead-based extraction technology, which allows for a standardized and efficient extraction process^29,30^. Also, we used droplet-digital PCR for absolute target copy quantification, a technology that is less prone to PCR inhibition than endpoint or qPCR^31,32^. Thus, we consider out methods robust to such biases.

Therefore, we assume the variation is due to biological factors, such as inter-individual differences in the digestive efficiency of the beetles^33^. Seed feeding in carabid beetles is facilitated by bacterial endosymbionts in guts, which can vary in their occurrence between individual beetles of the same species^34^, potentially affecting digestive capacity of individuals. It is possible that slight variation in size, age or maturation stage of the seeds we provided as food, could have affected copy number detections. However, to our knowledge, there is no literature available on the amount of chloroplast DNA in plant seeds.

It is possible that the regurgitated amount of gut content differed between individual beetles. Regurgitates are a non-lethal type of gut content sample^35^, that resembles whole-body extracts regarding the change in detection probability over time of digestion^36^. Regurgitation also occurs naturally in carabids: some species regurgitate the digestive enzymes contained in the crop, which is a part of the foregut, onto their prey for extra-oral digestion^37^. The crop of carabids is the part of the digestive system, where the most recent meal is stored and digested^37–39^. Therefore, it can be assumed that regurgitates are representative of the last meal consumed by the beetles. Sunderland^39^ found remains of previous meals in carabids’ hind guts. Thus, dissected guts as well as whole-body extracts are likely to contain DNA of the most recent meal and, additionally, of previous ones. However, for whole-body samples, the entire consumer is homogenized, leading to an overabundance of intact consumer DNA in the extract, potentially outcompeting the digested food remains during extraction. Dissected guts and regurgitates, even more so, contain less consumer tissue, thus an improved yield of food DNA is to be expected^29,36^. Whether extracts of entire digestive systems, such as in whole-bodies or dissected guts, differ from regurgitates regarding prey read numbers needs to be assessed.

It has been suggested that faeces are better suited for quantitative estimations than gut content samples because, as the final product of digestion, they are supposed to be more stable and, thus, directly comparable regarding the condition of target DNA^40^. However, as previously mentioned, the aptitude of prey DNA from faeces for quantitative metabarcoding is still debated^6,7^. Comparative studies actually suggest that gut content yields higher read counts in metabarcoding, potentially due to the more advanced degree of prey decomposition and the presence of inhibitory substances in faeces^41^. The disadvantage of gut content samples, however, is that the unknown time lapse since feeding in field-collected samples, can cause uncertainties for the estimation of ingested biomass. For a shotgun sequencing analysis, Paula et al.^42^ demonstrated how to estimate likely combinations of prey individual numbers consumed and time lapse since feeding based on experimentally determined DNA decay rates. Given there is little variation in expected DNA amounts per prey individual, and DNA digestion follows an exponential decay, the approach appears promising. However, neither of these two conditions applies to our present study system.

We found that the number of target copies/µl DNA extract derived from regurgitates was moderately correlated with the target plant read numbers recovered during high-throughput sequencing. PCR stochasticity is a frequently mentioned source of bias for the quantitative estimation from read numbers^43–45^, thus, the distortion between initial and final target fragment estimates did not come as a surprise. We implemented two check-points between ingested biomass and read numbers: droplet digital PCR for absolute copy number quantification and capillary electrophoresis for measuring the relative amount of the target PCR products. Our relative measure of the amount of PCR product, RFU-values, was moderately related to both, the initial copy numbers and the read numbers. Moreover, its correlation with either was higher than that observed among the two measures. As the RFU-values were a stronger predictor of the final read numbers than the initial copy numbers, we suggest optimizing the library preparation protocol in a way that RFU-values strongly reflect initial copy numbers. Our additional celPCR screenings of the samples, with 25, 30 and 40 cycles, revealed the best correlation between initial copy numbers and RFU-values after 30 cycles. This is in agreement with reports from pollen metabarcoding, where library preparation with 30 cycles improved the estimation of pollen grains from read numbers with *trn*L primers^46^. Moreover, Valentini^47^ recommend using 35 cycles at most when working with *trn*L primers to avoid the amplification of environmental DNA contaminations. Generally, high numbers of PCR cycles can lead to saturation, which results in a mismatch between RFU-values from celPCRs and absolute target copy numbers^23^. At the same time, too low cycle numbers have been found to impair estimations as well^44^. Correlations between copy number estimates and read numbers, or their respective proportions, have also been reported in studies using qPCR^48–50^. When implementing capillary electrophoresis in the HTS-library preparation workflow, we strongly recommend to do it after sample indexing or on subsamples only, to reduce the risk of cross-contamination. Based on our findings, we suggest that an adjustment of the overall cycle numbers used during library preparation can play a role in the degree of bias introduced into HTS data. While we think that protocol optimization through cycle number adjustment can increase the fit between copy and read counts, it will not reduce amplification biases due to, for example, primer mismatches^51^.

To study the detection of plant seed DNA in regurgitates of carabids, we targeted a conserved region in the chloroplast genome, the *trn*L P6 loop, which allows for successful amplification of a wide range of different plant taxa^50^, with the drawback of having a low taxonomic resolution^52^. Moreover, multi-locus pollen analysis suggests that plastid marker regions, such as *trn*L, are superior to nuclear markers regarding quantitative estimations from read counts^46,53^. The particular primer combination used in our study, which has highly conserved priming sites among land plants^54^, amplifies a rather short (~ 200 bp) PCR fragment. It has previously been used in a metabarcoding assay, resulting in a low taxonomic resolution, allowing primarily for family-level identification^55^, which was also true for our samples. In another study, the same primer combination has been used to target plants in a diagnostic multiplex PCR for the study of the diverse diet of omnivorous carabid beetles in arable land^56^.

Although the beetles in the feeding experiment were provided with seeds of only one plant species, the metabarcoding analysis yielded several reads of other plant families. Among the more abundant plant species, we found reads assigned to the family of *Poaceae*. As grass seeds are among the preferred seeds of *P. rufipes*^57^, the reads could stem from trace amounts of field-consumed seeds still contained in the beetles’ guts, despite the extensive digestion interval of at least 7 days prior to the feeding experiments. Also, the samples of the present experiment, were sequenced alongside other samples containing plant DNA. Therefore, it cannot be ruled out that the unexpected plant-family detections were derived through index switching. Index switching is a source of bias known to occur when combinatorial dual indexes are used for high-throughput sequencing with Illumina HiSeq and refers to the mis-assignment of indexes between libraries^58,59^. The use of unique dual indexes is a good solution to avoid this problem.

Relative estimation of copy numbers through RFU-values derived from celPCRs as demonstrated here, could also be implemented for diagnostic assays in gut content analysis. The high resolution of PCR product patterns separated by capillary electrophoresis facilitates the distinction between different fragment sizes also in multiplex diagnostic PCR approaches employing multiple group-specific primers^60^ or general primers with species-specific differences in amplicon size^52,61^. For both single- and multiplex diagnostic approaches, RFU-values have been found to correlate to target copy numbers^23^.

Here, we found that copy numbers and, further downstream, also read numbers, recovered from gut content samples showed a trend of decrease over time of digestion. However, recovered reads from individual samples may be difficult to use for biomass estimations, as there is a high inter-individual variation between samples. Internal standards or normalization could be a way to go about technical distortions, while biological factors that are part of digestion still need further investigation. For now, we recommend to use read number-derived estimates only with caution, and to plan metabarcoding experiments, based on gut content samples, to include as many replicate samples as possible.

## Material and methods

### Regurgitates

For the present study, we used gut content samples of adult carabid beetles of the species *P. rufipes*, collected in August 2014 and August 2015^25^. The carabids were collected with pitfall traps in arable land near Innsbruck/Austria. Adult beetles were kept individually in plastic cups (71 × 58 mm, height × diameter) with moist tissue paper, in a climate chamber at an artificial light regime of L:D 14:10 h and temperatures of 22 °C/12 °C (day/night). They were maintained on a diet of 1/3 mealworm (*Tenebrio molitor* L.) larva every fifth day until being starved for 5 days prior to the feeding experiments.

During the feeding experiment, the beetles were offered five seeds of *Taraxacum officinale* and a drop of water in fresh cups for 2 h in darkness. After feeding on at least one seed, beetles were left to digest without food for 0, 4, 8, 16, 24, 32, 48, 64, 72, 96 or 128 h. Per digestion time interval, 10–15 individuals were used for sample collection. Beetles were put individually into 1.5 ml microtubes and stimulated to regurgitate by dipping the tip of the tube briefly into hot water^62^. Each beetle was used for regurgitation only once during the entire experiment and set free afterwards.

For DNA extraction, regurgitates were mixed with 200 µl lysis buffer (200 µl 1 × TES, 5 µl Proteinase K (20 mg/ml), 1 mg Polyvinylpyrrolidone) and incubated at 58 °C for 3 h. DNA was extracted on a BioSprint^®^ 96 extraction platform (Qiagen, Hilden, Germany) with the DNA Blood and Tissue Kit (Qiagen) according to the manufacturer’s protocol with elusion in 200 µl 1 × TE buffer. The DNA extracts were stored at − 29 ± 1 °C before PCR testing.

### Primers

**Figure 5:**
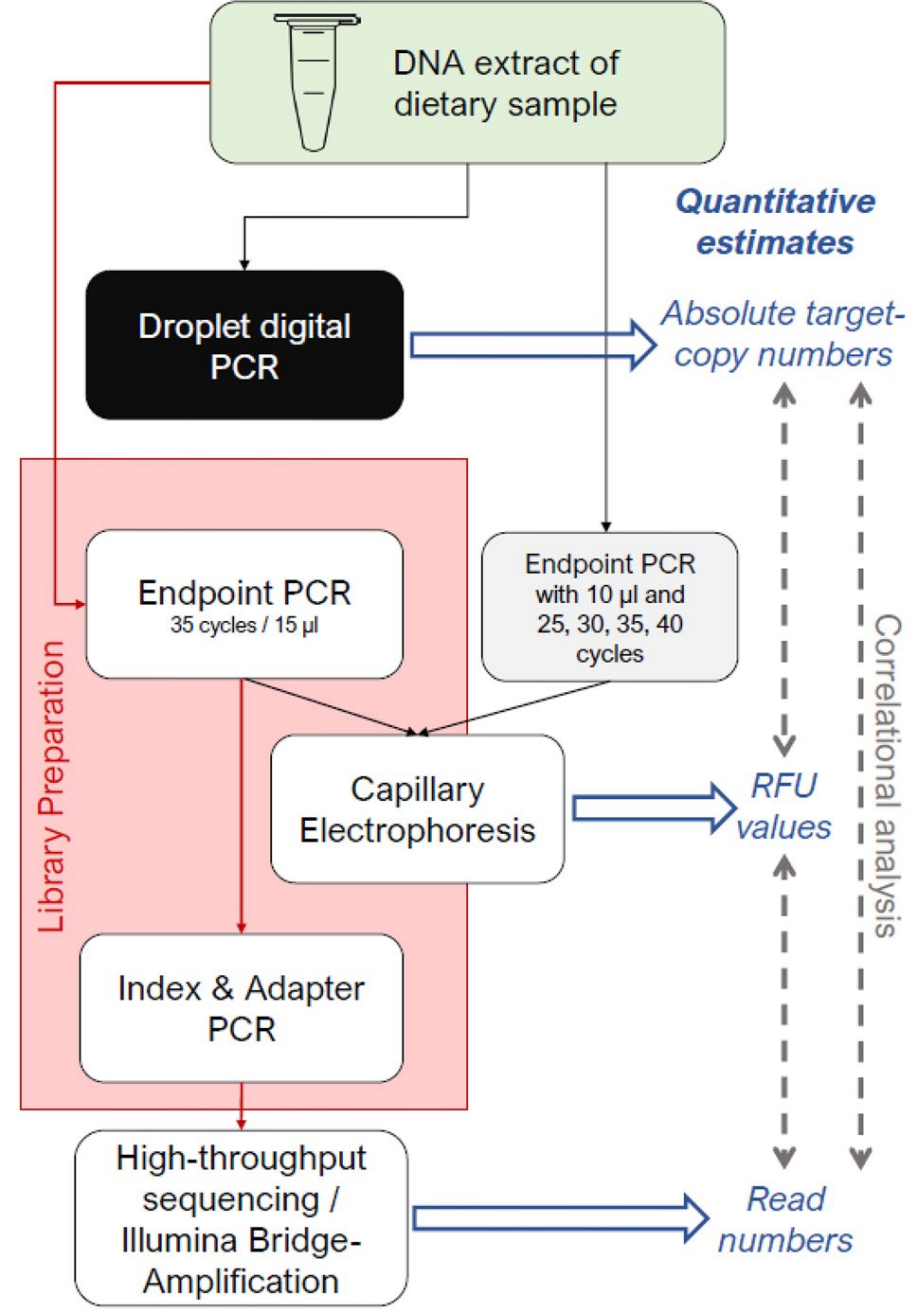
Flow diagram of analysis methods used on regurgitate samples of *Pseudoophonus rufipes* to get absolute and relative estimates of copy/read numbers contained in DNA extracts, PCR products and metabarcoding libraries. Measures of target DNA estimates at different analysis steps are indicated in blue.

For all PCR analysis (schematic overview in Fig. 5), we used the general plant primers *c* B49317 and* h* B49466^54,63^, which targets an approximately 200 bp long fragment of the *trn*L (UAA) region in the chloroplast genome^56^.

### Absolute quantification of target copy numbers with droplet digital (dd)PCR

The copies of the *trn*L *c-h* DNA fragment in DNA extracts of regurgitates were quantified by droplet digital PCR. Reaction mixes had a total volume of 22 µl, each containing 2.2 µl DNA template, 11.0 µl EvaGreen Supermix (Bio-Rad Laboratories, Inc., Hercules, CA, USA), 0.114 µM of each primer and 8.3 µl molecular grade water. Droplets were generated on the QX200 Automatic Droplet Generator System (Bio-Rad). Thermocycler conditions with optimized annealing temperature for the *trnL* primer pair were set according to the QX200 ddPCR EvaGreen Supermix user manual for 3-step PCRs. The final program started with 5 min of activation at 95 °C, followed by 40 cycles of 30 s denaturation at 95 °C, 1 min annealing at 58 °C and 1 min extension at 72 °C, followed by a first step of stabilization at 4 °C for 5 min and a second step at 90 °C for 5 min and finished with 12 °C on hold. Samples were analysed on the QX200 Droplet Digital™ System (Bio-Rad).

### celPCR: endpoint PCRs and capillary electrophoretic analysis

For endpoint PCRs, the 10 µl reaction volume contained 2.5 µl DNA template, 5.0 µl 2xType-it Multiplex PCR Master Mix (Qiagen), 0.5 µM of each primer, 0.5 µg, and 1.0 µl molecular grade water. Samples were screened in four PCR replicates, each with a different number of cycles without replications within each protocol. Thermocycling programs started with an initial denaturation for 5 min at 95 °C, followed by 25, 30, 35 or 40 cycles of 20 s denaturation at 92 °C, 90 s annealing at 55 °C and 60 s extension at 70 °C, and finished with a final elongation of 5 min at 70 °C. Samples that tested negative in the celPCRs with 40 and 35 cycles were not screened in celPCR-variants with 25 and 30 cycles and neither were they subject to target copy quantification in ddPCR or high-throughput sequencing (HTS). Batches of up to 94 samples were screened alongside one negative control and one positive control, containing molecular grade water or DNA of *T. officinale,* respectively.

PCR products were separated and visualized with a QIAxcel Advanced capillary electrophoresis system (Qiagen), using the process profile AM 320 with an injection time of 30 s. We set the parameters of the ScreenGel (Qiagen) software analysis profile to smoothing filter 15 pts, baseline 40 s, minimum 0.25 s, suspend integration 0.50 min, and a threshold 0.1 relative fluorescent units (RFU). Peaks below this threshold were manually inserted and measured.

### HTS-library preparation

64 samples testing positive for the *trn*L target amplicon were further analysed by high-throughput sequencing. Amplicon libraries were prepared with the Nextera^®^ XT Indices (Illumina, San Diego, CA, USA) in a combinatorial dual indexing strategy with a 2-step PCR protocol. In the first PCR, the general plant primers *trn*L *c-h* with appended adapter sequences, as described in the Nextera user manual, were used. The 15 µl reaction mix was prepared with 4 µl DNA template, 7.5 µl 2xType-it Multiplex PCR Master Mix (Qiagen), 0.5 µM of each primer, 0.33 µg BSA and 1.5 µl molecular grade water. Thermocycler conditions were set as described above for celPCRs with 35 cycles.

To remove primer dimers, 7 µl of undiluted PCR product were added to a mix of 0.1 µl Exonuclease I (10 u/µl) (NEB New England Biolabs, Ipswich, MA, USA), 0.1 µl rSAP (NEB) and 1.8 µl water and incubated for 15 min at 37 °C and 15 min at 80 °C in a thermocycler.

PCRs for attaching indices and sequencing adapters were done in total volumes of 25 µl for 5 µl cleaned PCR-1 product, 12.5 µl 2xType-it Multiplex PCR Master Mix (Qiagen), 1 µM of each adapter, 0.4 µg BSA and 1.5 µl water. Index-PCR conditions started with an initial denaturation at 95 °C for 5 min, followed by 8 cycles of denaturation at 95 °C for 30 s, annealing at 55 °C for 30 s and extension at 72 °C for 60 s, and final elongation at 72 °C for 5 min. Finally, libraries were cleaned with SPRIselect (Beckmann Coulter Inc., Brea, CA, US), following the manufacturer’s protocol for left side selection with a ratio of 1:0.8. Samples were pooled with equal volumes (10 µl per sample) and 7 µl of the final pool were sent for sequencing to the VBCF Vienna BioCenter Core Facilities (Vienna, Austria). Samples were sequenced on the Illumina MiSeq System (Illumina), PE300, with a reported total read number of 25.9 million reads/Q > 30 19.3; Bloomca PhiX (7.1).

### Bioinformatic processing of high-throughput sequencing data

High-throughput sequencing data were quality checked with FastQC (v0.11.8)^64^. Forward and reverse reads were extracted with samtools (v1.9, using htslib 1.9)^65^. Primers were removed with cutadapt (v3.5)^66^. Usearch (v11.0.667)^67^ was used for merging (settings: fastq_pc 90, fastq_maxdiffs 30) and quality filtering (settings: fastq_maxee 0.5) of paired-end reads, further, for identifying unique sequences, discarding singletons and sorting merged sequences by length, keeping only sequences with at least 130 bp. Finally, sequences were blasted with NCBI-blast + (v2.8.1)^68^ against the full NCBI nucleotide database (updated 29.10.2021). Sequences of organisms other than plants were discarded. Moreover, only sequences that matched the reference database at a level of 98% identity and above were used for further analysis.

### Data analysis and statistics

A total of 145 samples was initially screened for the target fragment in endpoint celPCRs with 40 and 35 cycles. Of these samples, 41 did not amplify DNA in either of the cycle-number variants, partly because of long digestion times, during which detection probability decreases. A total of 106 samples were additionally screened in PCRs with 25 and 30 cycles, and 0 RFU were assumed for samples that tested negative in the previous rounds of screening. We used 92 samples for measurement of copies/µl extract with ddPCR, and combined them with 40 samples, that were assumed to contain 0 copies because they repeatedly tested negative in celPCRs. Of the samples screened multiple times, 64 were further screened via high-throughput sequencing of the *trn*L c-h target region.

Statistical analysis was done with R statistical software (v3.5.0, 2018-04-23)^69^ and the plots were created using ggplot2^70^ with ggthemes^71^.

To study the change of copies/µl and read numbers of the target plant over time of digestion, we used only samples of beetles that had consumed three seeds during the feeding trials. The relationship of the copies and read numbers with the time of seed digestion by carabid beetles was assessed via the non-parametric Pearson correlation, because the data did not meet the assumptions for linear regression, even after data transformation. We used the Kruskal–Wallis test to test whether there were significant differences in copy numbers (and read) between different time points after feeding. To reveal which times differed significantly, we used the pairwise.wilcox function with the BH (Benjamini–Hochberg) correction, at the significance level of α = 0.05.

To study the correlation among the different quantitative measurements of the DNA fragment, we used all available samples, regardless of the numbers of seeds consumed but did not use the set of samples with “assumed 0 s”, as in the analysis over time. We assessed the relationship between initial copy numbers per µl DNA extract and reads or the signal strengths measured via capillary electrophoresis for end-point PCR-products (RFU-values) with linear regression models. Therefore, were used the log-transformed copy numbers (natural log (copies + 1)) as the response variable and reads or RFU-values as the predictors. Model assumptions were tested through visual inspection of residual plots and by applying the ‘ols_test_normality’-function of the package olsrr^72^ to test for normality and the function ‘ncvTest’ (Non-Constant Error Variance or Breusch-Pagan test) included in the car package^73^ to test for heteroscedasticity. If the assumption of linearity was not met, RFU-values were log-transformed as well. For all models, we calculated robust standard errors with ‘coeftest’ in the lmtest package^74^ combined with the function ‘vcovHC’ of the sandwich package^75,76^ and bootstrapped confidence intervals, using the functions ‘boot’ and ‘boot.ci’ of the boot package^77,78^.

## Supplementary Information

Supplementary Information.

## Data Availability

Original data of droplet digital PCRs, celPCRs and high-throughput sequencing are available at http://doi.org/10.17605/OSF.IO/C49N3.
