# Supplementary material for: Laboratory protocol is important to improve the correlation between target copies and metabarcoding read numbers of seed DNA in ground beetle regurgitates
Source: Sci Rep. 2023 Feb 3;13:1995. doi: 10.1038/s41598-023-29019-8 (PMC9898267; doi:10.1038/s41598-023-29019-8)
Supplement: Supplementary file 1 — Supplementary Information. [file 41598_2023_29019_MOESM1_ESM.docx]

**SUPPLEMENTARY INFORMATION**

**Laboratory protocol is important to improve the correlation between target copies and metabarcoding read numbers of seed DNA in ground beetle regurgitates**

Veronika Neidel*, Michael Traugott

*Applied Animal Ecology Research Unit, Department of Zoology, University of Innsbruck, Technikerstraße 25, 6020 Innsbruck, Austria.*

Correspondence:

*E-Mail: [Veronika.Neidel@uibk.ac.at](mailto:Veronika.Neidel@uibk.ac.at)

Supplementary Table S1: Results of linear regressions analysing the relationship of the metabarcoding read numbers with (A) the copies/µl DNA extract and (B) the signal strength measured in RFU values of the library-preparation PCR products of the *trn*L c-h target fragment in regurgitates of *Pseudoophonus rufipes* at 0, 4, 8, 16, 24, 48, 72 and 96 after consumption of 1-3 seeds of *T. officinale.*

|  |  |  |  | **robust** | | **Bootstrapped C**I | |  |  | |  |  |
| --- | --- | --- | --- | --- | --- | --- | --- | --- | --- | --- | --- | --- |
|  | **response ~ predictor** | **Mult. R²** | **coefficient** | **estimate** | **SE** | **lower**  **95% CI** | **upper 95% CI** | **t-value** | **p-value** | | **σ** | **df** |
|  |  |  |  |  |  |  |  |  |  | |  |  |
| **(A)** | **Asteracaea reads ~ log(copies + 1)** | 0.429 | *Intercept* | 5009.000 | 2844.400 | 531.000 | 12569.000 | 1.761 | 0.083 | . | 13330 | 62 |
|  |  |  | *log(copies + 1)* | 5348.500 | 864.300 | 3309.000 | 6822.000 | 6.188 | 0.000 | *** |  |  |
|  |  |  |  |  |  |  |  |  |  |  |  |  |
| **(B)** | **Asteracaea reads ~ RFU_i35** | 0.536 | *Intercept* | 7513.420 | 1911.170 | 4462.000 | 12262.000 | 3.931 | 0.000 | *** | 12020 | 62 |
|  |  |  | *RFU_i35* | 5534.450 | 792.530 | 3959.000 | 6977.000 | 6.983 | 0.000 | *** |  |  |

Supplementary Table S2: Results of the linear regression of target copies/µl (log(copies/µl +1)) modelled with signal strengths of celPCR products [RFU] derived from (A) the HTS library preparation PCR with 35 cycles and a 15 µl reaction volume, or (B-E) PCRs with 25, 30, 35 or 40 cycles and a 10 µl reaction volume. Copies/µl DNA extract of the plant specific target fragment t*rnL c-h* were amplified in regurgitate samples collected from *Pseudoophonus rufipes at* 0, 4, 8, 16, 24, 48, 72, 96 or 128 h after feeding on 1-3 seeds of *Taraxacum officinale*. Residual standard error and degrees of freedom are reported in the columns σ and df.

|  |  |  |  | **robust** | | **Bootstrapped C**I | |  |  |  |  |  |
| --- | --- | --- | --- | --- | --- | --- | --- | --- | --- | --- | --- | --- |
|  | **cycle number** | **Mult. R²** | **coefficient** | **estimate** | **SE** | **lower 95% CI** | **upper 95% CI** | **t-value** | **p-value** | | **σ** | **df** |
| **(A)** | **RFU i35** | 0.588 | Intercept | 0.923 | 0.191 | 0.641 | 1.463 | 4.830 | < 0.001 | *** | 1.464 | 86 |
|  |  |  | log(RFU_i35+1) | 0.768 | 0.076 | 0.602 | 0.915 | 10.085 | < 0.001 | *** |  |  |
|  |  |  |  |  |  |  |  |  |  | |  |  |
| **(B)** | **RFU 25** | 0.741 | Intercept | 8.843 | 0.182 | 8.473 | 9.199 | 48.521 | < 0.001 | *** | 1.157 | 90 |
|  |  |  | log(RFU_25 + 0.001) | 1.105 | 0.043 | 1.016 | 1.186 | 25.878 | < 0.001 | *** |  |  |
| **(C)** | **RFU 30** | 0.951 | Intercept | 5.694 | 0.103 | 5.520 | 5.943 | 55.107 | < 0.001 | *** | 0.502 | 90 |
|  |  |  | log(RFU_30 + 0.01) | 1.163 | 0.030 | 1.111 | 1.230 | 38.765 | < 0.001 | *** |  |  |
| **(D)** | **RFU 35** | 0.849 | Intercept | 0.428 | 0.109 | 0.266 | 0.729 | 3.941 | < 0.001 | *** | 0.886 | 90 |
|  |  |  | RFU_35 | 1.766 | 0.092 | 1.581 | 1.942 | 19.225 | < 0.001 | *** |  |  |
| **(E)** | **RFU 40** | 0.538 | Intercept | -0.722 | 0.254 | -1.290 | -0.267 | -2.837 | 0.006 | ** | 1.546 | 90 |
|  |  |  | RFU_40 | 1.177 | 0.106 | 0.986 | 1.407 | 11.114 | < 0.001 | *** |  |  |

Supplementary Table S3: Mean copy number/µl measured with droplet digital PCR in the regurgitates of *Pseudoophonus rufipes* after feeding on 1-3 seeds of *T. officinale*. Mean, standard deviation and sample numbers are presented for the dataset with and without the samples assumed to contain 0 copies due to being negative for the *trn*L c-h target fragment in repeated screening with celPCRs

|  | **measured values only** | | | | **with assumed 0s** | | | |
| --- | --- | --- | --- | --- | --- | --- | --- | --- |
|  | **copies / µl** | | | **samples** | **copies / µl** | | | **samples** |
| **digestion time [h]** | **mean** | **±** | **sd** | **n** | **mean** | **±** | **sd** | **n** |
| 0 | 372.3 | **±** | 932.1 | 10 | 372.3 | **±** | 932.1 | 10 |
| 4 | 515.5 | **±** | 1508.3 | 13 | 418.9 | **±** | 1365.0 | 16 |
| 8 | 214.6 | **±** | 598.5 | 9 | 175.6 | **±** | 542.3 | 11 |
| 16 | 70.4 | **±** | 137.1 | 11 | 59.6 | **±** | 127.9 | 13 |
| 24 | 182.6 | **±** | 345.2 | 10 | 152.1 | **±** | 320.2 | 12 |
| 32 | 35.9 | **±** | 84.1 | 9 | 27.0 | **±** | 73.5 | 12 |
| 48 | 36.4 | **±** | 58.2 | 7 | 25.5 | **±** | 50.7 | 10 |
| 64 | 16.9 | **±** | 35.6 | 7 | 10.7 | **±** | 28.8 | 11 |
| 72 | 2.3 | **±** | 4.2 | 7 | 1.1 | **±** | 3.1 | 14 |
| 96 | 11.2 | **±** | 17.0 | 5 | 4.3 | **±** | 11.4 | 13 |
| 128 | 0.5 | **±** | 0.7 | 4 | 0.2 | **±** | 0.5 | 10 |

Supplementary Table S4: Mean number of reads of the family *Asteraceae,* detected in high-throughput sequencing of regurgitate samples of *Pseudoophonus rufipes* after feeding on 1-3 seeds of *T. officinale*. Mean, standard deviation and sample numbers are presented for the dataset with and without the samples assumed to contain 0 copies due to being negative for the *trn*L c-h target fragment in repeated screening with celPCRs

|  | **measured values only** | | | | **with assumed 0s** | | | |
| --- | --- | --- | --- | --- | --- | --- | --- | --- |
|  | **reads** | | | **samples** | **reads** | | | **samples** |
| **digestion time [h]** | **mean** | **±** | **sd** | **n** | **mean** | **±** | **sd** | **n** |
| 0 | 413.67 | **±** | 978.86 | 9 | 372.30 | **±** | 932.10 | 10 |
| 4 | 595.86 | **±** | 1720.91 | 10 | 425.61 | **±** | 1458.87 | 14 |
| 8 | 214.62 | **±** | 598.52 | 9 | 175.60 | **±** | 542.33 | 11 |
| 16 | 110.42 | **±** | 161.84 | 7 | 70.27 | **±** | 137.18 | 11 |
| 24 | 364.59 | **±** | 430.44 | 5 | 202.55 | **±** | 359.95 | 9 |
| 32 | 40.43 | **±** | 88.75 | 8 | 26.96 | **±** | 73.55 | 12 |
| 48 | 51.01 | **±** | 64.49 | 5 | 25.50 | **±** | 50.71 | 10 |
| 64 | 29.35 | **±** | 45.21 | 4 | 11.74 | **±** | 30.19 | 10 |
| 72 | 3.98 | **±** | 5.18 | 4 | 1.14 | **±** | 3.11 | 14 |
| 96 | 18.67 | **±** | 19.28 | 3 | 4.31 | **±** | 11.36 | 13 |
| 128 | - | **±** | - | - | 0.00 | **±** | 0.00 | 8 |

Supplementary Table S5: Results of the non-parametric Kruskal-Wallis test and the follow-up pairwise comparison with Wilcoxon tests with BH correction, revealing differences in (a) the copy numbers (n=111) and, (b) the read numbers (n=102) between samples collected at different times after feeding. Regurgitates of beetles were collected at 0, 4, 8, 16, 24, 48, 72, 96 and 128 after consumption of three seeds. Datasets include samples that were assumed to contain 0 target fragments due to repeatedly testing negative for the target *trn*L c-h fragment in celPCRs.

| 1. **copies** | | **Kruskal-Wallis chi-squared = 31.187, df = 10, p-value = 0.0005464** | | | | | | | | | |
| --- | --- | --- | --- | --- | --- | --- | --- | --- | --- | --- | --- |
|  |  | **digestion time** | | | | | | | | | |
|  |  | **0** | **4** | **8** | **16** | **24** | **32** | **48** | **64** | **72** | **96** |
| **digestion time** | **4** | 0.434 |  |  |  |  |  |  |  |  |  |
|  | **8** | 0.3 | 0.778 |  |  |  |  |  |  |  |  |
|  | **16** | 0.269 | 0.521 | 0.352 |  |  |  |  |  |  |  |
|  | **24** | 0.3 | 0.673 | 0.473 | 0.821 |  |  |  |  |  |  |
|  | **32** | 0.166 | 0.434 | 0.280 | 0.942 | 0.824 |  |  |  |  |  |
|  | **48** | 0.193 | 0.352 | 0.259 | 0.557 | 0.521 | 0.773 |  |  |  |  |
|  | **64** | 0.198 | 0.414 | 0.239 | 0.560 | 0.508 | 0.56 | 0.778 |  |  |  |
|  | **72** | 0.035 | 0.047 | 0.019 | 0.166 | 0.184 | 0.192 | 0.436 | 0.773 |  |  |
|  | **96** | 0.019 | 0.035 | 0.019 | 0.09 | 0.084 | 0.166 | 0.289 | 0.778 | 0.915 |  |
|  | **128** | 0.068 | 0.073 | 0.025 | 0.213 | 0.194 | 0.194 | 0.562 | 0.824 | 0.945 | 0.778 |

| 1. **reads** | | **Kruskal-Wallis chi-squared = 34.689, df = 10, p-value = 0.0001411** | | | | | | | | | |
| --- | --- | --- | --- | --- | --- | --- | --- | --- | --- | --- | --- |
|  |  | **digestion time** | | | | | | | | | |
|  |  | **0** | **4** | **8** | **16** | **24** | **32** | **48** | **64** | **72** | **96** |
| **digestion time** | **4** | 0.55 |  |  |  |  |  |  |  |  |  |
|  | **8** | 0.692 | 0.642 |  |  |  |  |  |  |  |  |
|  | **16** | 0.209 | 0.523 | 0.360 |  |  |  |  |  |  |  |
|  | **24** | 0.213 | 0.597 | 0.336 | 1.000 |  |  |  |  |  |  |
|  | **32** | 0.082 | 0.322 | 0.132 | 0.583 | 0.832 |  |  |  |  |  |
|  | **48** | 0.094 | 0.336 | 0.133 | 0.583 | 0.799 | 0.88 |  |  |  |  |
|  | **64** | 0.11 | 0.213 | 0.096 | 0.250 | 0.447 | 0.261 | 0.550 |  |  |  |
|  | **72** | 0.019 | 0.07 | 0.019 | 0.110 | 0.162 | 0.132 | 0.250 | 1.000 |  |  |
|  | **96** | 0.02 | 0.065 | 0.019 | 0.094 | 0.213 | 0.132 | 0.25 | 1.000 | 1.000 |  |
|  | **128** | 0.049 | 0.091 | 0.03 | 0.094 | 0.149 | 0.091 | 0.149 | 0.437 | 0.447 | 0.428 |

Supplementary Table S6: Absolute sequence read numbers and proportion of total assigned reads for different plant families identified over all sequenced samples. Data for sequences of a fragment length of 135-160 bp.

| **Plant family** | **Total sequence reads** | **Percent of**  **Identified reads** |
| --- | --- | --- |
| Asteraceae | 1369082 | 87.62 |
| Poaceae | 56376 | 3.61 |
| Rosaceae | 33534 | 2.15 |
| Sapindaceae | 32855 | 2.10 |
| Solanaceae | 22432 | 1.44 |
| Fabaceae | 17940 | 1.15 |
| Cactaceae | 15262 | 0.98 |
| Euphorbiaceae | 5544 | 0.35 |
| Cornaceae | 2455 | 0.16 |
| Adoxaceae | 2453 | 0.16 |
| Brassicaceae | 2147 | 0.14 |
| Calyceraceae | 1172 | 0.08 |
| Talinaceae | 490 | 0.03 |
| Plantaginaceae | 485 | 0.03 |
| Amaryllidaceae | 93 | 0.01 |
| Lamiaceae | 59 | 0.00 |
| Rubiaceae | 35 | 0.00 |
| Pedaliaceae | 20 | 0.00 |
| Cucurbitaceae | 10 | 0.00 |
| Chenopodiaceae | 7 | 0.00 |
| Ranunculaceae | 4 | 0.00 |
| Amaranthaceae | 2 | 0.00 |
| Nyssaceae | 2 | 0.00 |


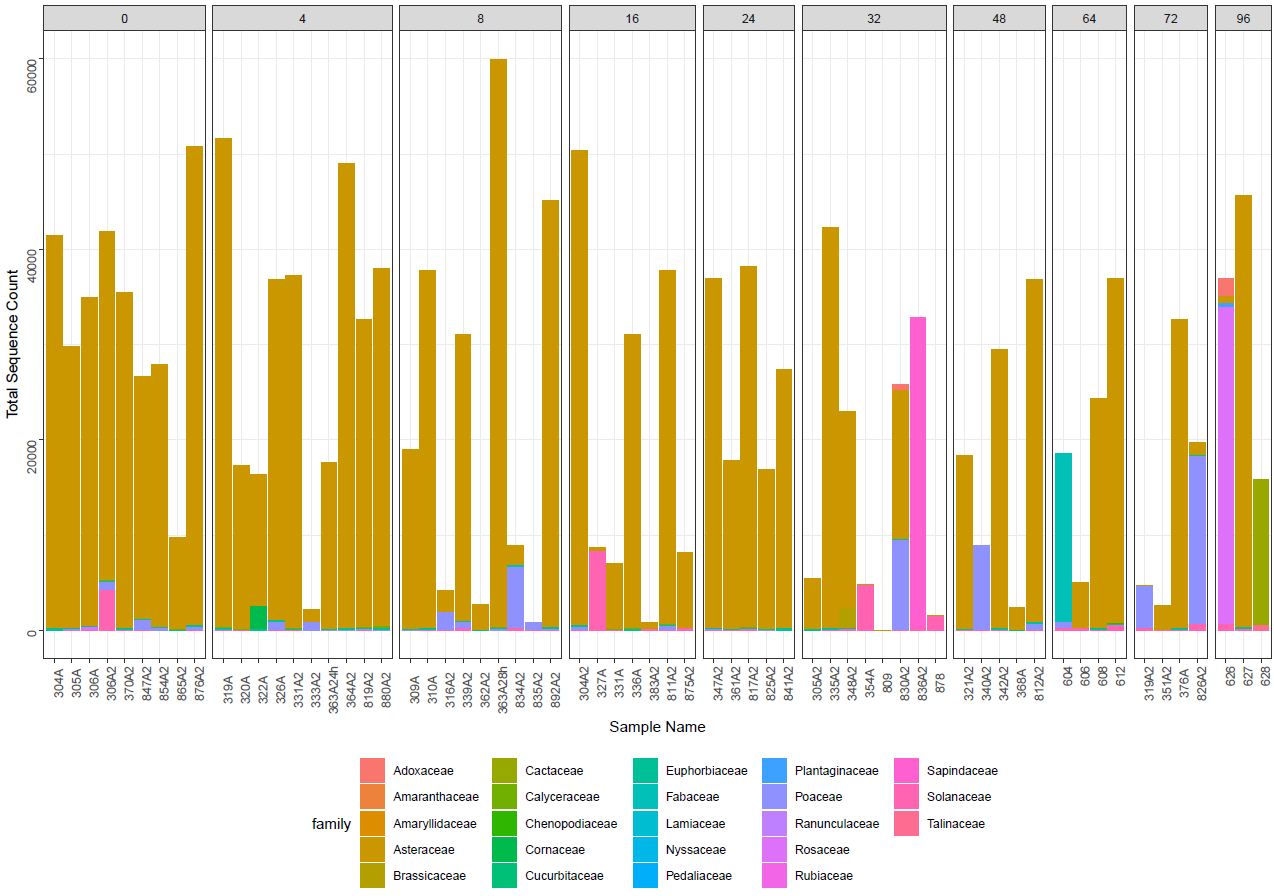


Supplementary Figure S1: Number of reads per sample assigned to different plant families. Data for sequences of a fragment length of 135-160 bp.
